# Application of an original RT-PCR–ELISA multiplex assay for MDR1 and MRP, along with p53 determination in node-positive breast cancer patients

**DOI:** 10.1054/bjoc.1999.0896

**Published:** 1999-12-08

**Authors:** J M Ferrero, M C Etienne, J L Formento, M Francoual, P Rostagno, I Peyrottes, F Ettore, E Teissier, P Leblanc-Talent, M Namer, G Milano

**Affiliations:** Oncopharmacology Unit, Centre Antoine Lacassagne, 33 Avenue de Valombrose, Nice Cedex 2, 06189, France

**Keywords:** p53, MDR1, MRP, breast cancer, multiplex assay

## Abstract

The long-term prognostic value of tumoural MDR1 and MRP, along with p53 and other classical parameters, was analysed on 85 node-positive breast cancer patients receiving anthracycline-based adjuvant therapy. All patients underwent tumour resection plus irradiation and adjuvant chemotherapy (the majority receiving fluorouracil–epirubicin–cyclophosphamide). Median follow-up for the 54 alive patients was 7.8 years. Mean age was 53.7 years (range 28–79) and 54 patients were post-menopausal. MDR1 and MRP expression were quantified according to an original reverse transcription polymerase chain reaction multiplex assay with colourimetric enzyme-linked immunosorbent assay detection(β2-microglobulin as control). P53 protein was analysed using an immunoluminometric assay (Sangtec). MDR1 expression varied within an 11-fold range (mean 94, median 83), MRP within a 45-fold range (mean 315, median 242) and p53 protein from the limit of detection (0.002 ng mg^−1^) up to 35.71 ng mg^−1^(mean 1.18, median 0.13 ng mg^−1^). P53 protein was significantly higher in oestrogen receptor (ER)-negative than in ER-positive tumours (*P* = 0.039). The higher the p53, the lower the MDR1 expression (*P* = 0.015, *r* = –0.27). P53 was not linked to progesterone receptor (PR) status, S phase fraction, or MRP. Significantly greater MDR1 expression was observed in grade I tumours (*P* = 0.029). No relationship was observed between MDR1 and MRP. Neither MDR1 nor MRP was linked to ER or PR status. Unlike MDR1, MRP was correlated with the S phase: the greater the MRP, the lower the S phase (*P* = 0.006, *r* = –0.42). Univariate Cox analyses revealed that MDR1, MRP, p53 and S phase had no significant influence on progression-free or specific survival. A tendency suggested that the greater the p53, the shorter the progression-free survival (*P* = 0.076 as continuous and 0.069 as dichotomous). © 2000 Cancer Research Campaign


*British Journal of Cancer* (2000) **82**(1), 171–177
© 2000 Cancer Research Campaign
Article no. bjoc.1999.0896

### Application of an original RT-PCR–ELISA multiplex assay for MDR1 and MRP, along with p53 determination in node-positive breast cancer patients

**JM Ferrero, MC Etienne, JL Formento, M Francoual, P Rostagno, I Peyrottes, F Ettore, E Teissier, P Leblanc-Talent, M Namer and G Milano**

Centre Antoine Lacassagne, Oncopharmacology Unit, 33 Avenue de Valombrose, 06189 Nice Cedex 2, France

**Summary** The long-term prognostic value of tumoural MDR1 and MRP, along with p53 and other classical parameters, was analysed on 85 node-positive breast cancer patients receiving anthracycline-based adjuvant therapy. All patients underwent tumour resection plus irradiation and adjuvant chemotherapy (the majority receiving fluorouracil–epirubicin–cyclophosphamide). Median follow-up for the 54 alive patients was 7.8 years. Mean age was 53.7 years (range 28–79) and 54 patients were post-menopausal. MDR1 and MRP expression were quantified according to an original reverse transcription polymerase chain reaction multiplex assay with colourimetric enzyme-linked immunosorbent assay detection (β2-microglobulin as control). P53 protein was analysed using an immunoluminometric assay (Sangtec). MDR1 expression varied within an 11-fold range (mean 94, median 83), MRP within a 45-fold range (mean 315, median 242) and p53 protein from the limit of detection (0.002 ng $mg^{-1}$) up to 35.71 ng $mg^{-1}$ (mean 1.18, median 0.13 ng $mg^{-1}$). P53 protein was significantly higher in oestrogen receptor (ER)-negative than in ER-positive tumours ($P$ = 0.039). The higher the p53, the lower the MDR1 expression ($P$ = 0.015, $r$ = –0.27). P53 was not linked to progesterone receptor (PR) status, S phase fraction, or MRP. Significantly greater MDR1 expression was observed in grade I tumours ($P$ = 0.029). No relationship was observed between MDR1 and MRP. Neither MDR1 nor MRP was linked to ER or PR status. Unlike MDR1, MRP was correlated with the S phase: the greater the MRP, the lower the S phase ($P$ = 0.006, $r$ = –0.42). Univariate Cox analyses revealed that MDR1, MRP, p53 and S phase had no significant influence on progression-free or specific survival. A tendency suggested that the greater the p53, the shorter the progression-free survival ($P$ = 0.076 as continuous and 0.069 as dichotomous). © 2000 Cancer Research Campaign

**Keywords:** p53; MDR1; MRP; breast cancer; multiplex assay

Numerous molecular markers have been investigated by means of univariate or multivariate analyses aimed at predicting breast cancer patient outcome (Gasparini et al, 1993). So far, the majority of such multivariate studies have been conducted in node-negative patients in order to identify subgroups of patients which could benefit from adjuvant treatment. In contrast, most node-positive breast cancer patients systematically received adjuvant chemotherapy. In support of this strategy, a recent meta-analysis performed on 30 000 early breast cancer patients demonstrated that adjuvant polychemotherapy (versus no chemotherapy) significantly improved disease-free and overall survival; moreover, it was suggested that anthracycline-containing regimens were associated with greater efficiency as compared to cyclophosphamide–methotrexate–fluorouracil (Early Breast Cancer Trialists' Collaborative Group, 1998). Since chemotherapy is known to impair quality of life, identification of prognostic markers in node-positive patients should be undertaken to avoid ineffective adjuvant therapy in intrinsically resistant tumours. It has been widely demonstrated that breast cancer tumours that are positive for oestrogen receptors (ER) benefit most from hormonal treatment (Early Breast Cancer Trialists' Collaborative Group, 1992). Among tumoural parameters potentially useful to predict responsiveness to chemotherapy, one can single out factors intrinsically related to the drug's mechanisms of action. Among the latter is the expression of c-*erb*-B2, reported to be a marker of responsiveness to high-dose adjuvant chemotherapy in node-positive breast cancer patients (Muss et al, 1994). Also, a low tumoural concentration of the lysosomal protease cathepsin D has been significantly related to longer survival in node-positive breast cancer patients receiving adjuvant chemotherapy (Namer et al, 1991). It has been established that defects in apoptosis caused by the inactivation of p53 tumour suppressor gene can produce treatment-resistant tumours, suggesting that p53 status may be an important determinant of tumour response to anticancer drugs (Lowe et al, 1994). Among factors more closely related to the drug mechanisms of action, the expression of MDR1 (Pastan and Gottesman, 1987) and, more recently, MRP (Barrand et al, 1994) are particularly relevant for predicting the sensitivity to anthracyclines, which are still held as reference drugs in breast cancer treatment.

Our purpose was to develop an original reverse transcription polymerase chain reaction (RT-PCR)–enzyme-linked immunosorbent assay (ELISA) multiplex assay for the coupled analysis of MDR1 and MRP. This assay was applied in 85 node-positive breast tumours from patients receiving anthracycline-based adjuvant therapy. A long-term prognostic analysis including p53, MDR1, MRP and other more classical prognostic factors was performed with a median follow-up of 7.8 years.

*Received 10 February 1999*
*Revised 4 June 1999*
*Accepted 2 July 1999*

*Correspondence to:* G Milano. E-mail: gerard.milano@cal.nice.fnclcc.fr

### MATERIALS AND METHODS

### Patients

Node-positive breast cancer patients were selected from an updated computerized database according to the following criteria: patients classified as node-positive (one node involved or more); patients having received anthracycline-based adjuvant therapy; patients followed up at our institute; patients with sufficient remaining tumour material to assay MDR, MRP and p53. This retrospective study was thus conducted on 85 patients. A description of the population is given in Table 1. Mean age was 53.7 years (range 28–79). Fifty-four patients out of 85 were post-menopausal. The histological grade, scored according to previously published classifications (Bloom and Richardson, 1957; Scarff and Torloni, 1968), was not performed on the nine patients with lobular or colloid carcinoma. Determination of the S phase fraction (flow cytometry) was available in 41 patients. Cytosolic ERs and progesterone receptors (PRs) were assayed by an immunoassay performed with the Abbott Kit (Romain et al, 1994). Thresholds for positivity were 10 and 15 fmol $mg^{-1}$ prot for ER and PR respectively.

All selected patients had undergone complete tumour resection with axillary lymph node dissection. The mean number of involved nodes was 5.4 (median 4.0, range 1–35). All patients received post-operative irradiation and adjuvant chemotherapy. The chemotherapy protocol was FEC (fluorouracil–epirubicin–cyclophosphamide) in 67 patients; FAC (fluorouracil–adriamycin–cyclophosphamide) in 12 patients; epirubicin alone in five patients; and AECF (adriamycin–vindesine–cyclophosphamide–fluorouracil) in one patient. In addition, 37 patients received tamoxifen, four received luteinizing hormone releasing hormone (LHRH) and two underwent castration. All patients were regularly followed up with clinical, radiological and biological examinations every 6 months for the first 5 years and yearly examinations thereafter.

**Table 1** Description of the population

| | Number of patients | % |
|---|---|---|
| Node involvement | | |
| 1–3 nodes | 42 | 49.4 |
| 4–7 nodes | 20 | 23.5 |
| 8–35 nodes | 23 | 27.1 |
| Tumour size | | |
| T1 | 14 | 17.1 |
| T2 | 54 | 65.9 |
| T3 | 12 | 14.6 |
| T4 | 2 | 2.4 |
| Histological grade | | |
| I | 22 | 25.9 |
| II | 35 | 41.2 |
| III | 19 | 22.3 |
| Not scored | 9 | 10.6 |
| Positive receptor status | | |
| ER | 61 | 71.8 |
| PR | 56 | 65.9 |

[a]Tumour size was unknown for three patients.

### MDR1-MRP analysis

#### *RNA extraction and RT*

MDR1 and MRP were assayed on a tumoural fragment stored in liquid nitrogen. Total RNA was isolated using the RNA NOW kit from Biogentex (Ozyme, Montigny-le-Bretonneux, France) based on a method derived from Chomczynski and Sacchi (1987). RNA quality was checked by agarose gel electrophoresis. Quantification was performed by densitometric analysis at 260 nm. One microgram of total RNA was preincubated for 5 min at 65°C in a 20 μl final volume of 50 mM Tris–HCl (pH 8.3), 75 mM potassium chloride (KCl), 3 mM magnesium chloride ($MgCl_2$), 1 mM of each deoxyribonucleotide triphosphate and 2 μM of random hexamers (Roche Diagnostics, Meylan, France). Fifty units of Expand Reverse Transcriptase (Roche Diagnostics) and 20 units of human placenta ribonuclease inhibitor (Amersham Pharmacia Biotech, les Ulis, France) were then added and the mixture was incubated for 30 min at 42°C followed by 5 min at 94°C.

#### *Primers*

The oligonucleotides used for MDR1 amplification were: MDR1 sense-strand: CCC ATC ATT GCA ATA GCA GG (nt. 2596–2615) and MDR1 antisense-strand: GTT CAA ACT TCT GCT CCT GA (nt. 2733–2752), which yield a 167 bp product (Noonan et al, 1990).

For MRP amplification, oligonucleotides were: MRP sense-strand: GAC CTG GAC TTC GTT CTC A (nt. 4109–4127) and MRP antisense-strand: ACG TCC AGA TTC CTT CAT CC (nt. 4381–4400), which yield a 291 bp product (Abbaszadegan et al, 1994; slighty modified).

Those used for amplification of the reference gene ($\beta_2$-microglobulin) were: $\beta_2$ $\mu_3$ sense-strand: ACC CCC ACT GAA AAA GAT GA (nt. 308–327) and $\beta_2$ $\mu_4$ antisense-strand: ATC TTC AAA CCT CCA TGA TG (nt. 402–421), which yield a 120 bp product (Noonan et al, 1990).

All primer pairs span an intron to distinguish the PCR products generated from cDNA and genomic DNA.

Three specific capture probes, 5′biotinylated and purified by high performance liquid chromatography (HPLC; Eurobio, les Ulis, France) and corresponding to each amplification product, were used for ELISA detection: MDR1 capture probe: GAA AAT GTT GTC TGG ACA AGC (nt. 2628–2648); MRP capture probe: GGG CTT ATT TCG GAT CAA CG (nt. 4210–4229); $\beta_2$-microglobulin capture probe: GTG GGA TCG AGA CAT GTA AG (nt. 379–398).

#### *PCR conditions*

Briefly, 250 ng RNA equivalent were subjected to PCR amplification in a 100 μl final volume containing 10 mM Tris–HCl (pH 8.3), 50 mM KCl, 1.5 mM $MgCl_2$, 200 μM of each deoxyribonucleotide triphosphate (dATP, dCTP, dGTP), 190 μM of dTTP, 10 μM of dUTP labelled with digoxigenin, 2.5 units of *Taq* polymerase and 250 nM of each primers pair for MDR1, MRP and $\beta_2$-microglobulin. The multiplex amplification consisted of an initial 5-min incubation at 94°C followed by 22 amplification cycles (94°C for 30 s, 55°C for 30 s and 72°C for 30 s).

#### *PCR ELISA*

MDR1 and MRP amplifications were performed using the PCR-ELISA digoxigenin (DIG) labelling and the PCR-ELISA DIG detection kits (Roche Diagnostics, Meylan, France) as previously described by us (Castillo et al, 1997). The principle of PCR ELISA is presented in Figure 1. The DIG-labelling reaction of the PCR

 © 2000 Cancer Research Campaign

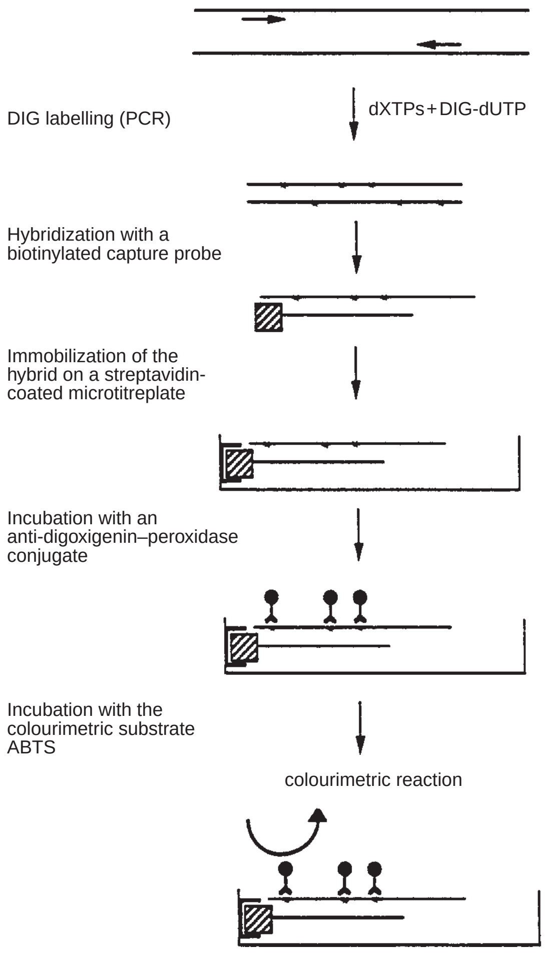

**Figure 1** Principles of PCR ELISA

products was carried out during co-amplification of MDR1, MRP and $\beta_2$-microglobulin for an optimal number of cycles, in the presence of digoxigenin-labelled dUTP. These labelled products were analysed with the three specific biotinylated capture probes which allowed immobilization of the hybrid to a streptavidin-coated microplate surface. The bound hybrid was detected by an anti-digoxigenin antibody–peroxidase conjugate. Peroxidase activity was evaluated by addition of the colourimetric substrate ABTS and the absorbance was read at 405 nm. Results were arbitrarily expressed as 10 000–fold the absorbance ratio (MDR1 or MRP/$\beta_2$-microglobulin).

### P53 analysis

The cytosolic p53 protein (wild-type and mutated forms) was analysed on a tumoural cytosol stored in liquid nitrogen, using a monoclonal two-site single incubation immunoluminometric assay (LIA-mat, Sangtec, Sweden). The sensitivity limit was 0.002 ng $mg^{-1}$ prot. Cytosolic proteins were determined by the Bradford colourimetric technique (Biorad Laboratories GmbH, Munich, Germany). The intra-assay ($n$ = 5) and inter-assay ($n$ = 5) reproducibility were 7% and 9.5% respectively.

### Statistics

Gaussian distribution was evaluated according to normal probability plot and Kolmogorov–Smirnov good-fit test. Since p53, MDR1, MRP and S phase fraction did not fit a Gaussian distribution, relationships between tumoural parameters were analysed using non-parametric tests. Duration of survival was calculated from the date of surgery. For specific survival, the end point was breast cancer-related death. For progression-free survival, the end point was either recurrence or metastasis. Survival curves were computed using the Kaplan–Meier method. Two patients were lost to follow-up and were considered as censored observations. At time of analysis, 31 patients had died. Median follow-up was 83 months for the whole population and 94 months for alive patients. The influence of tumoural parameters on specific and progression-free survival was analysed according to the Cox proportional hazard regression, using logarithm 10-transformed data for S phase fraction, p53, MDR1 and MRP. Statistics were drawn up on SPSS software (Chicago, IL, USA).

### RESULTS

### Characteristics of the RT-PCR–ELISA multiplex assay

Densitometric analysis showed that the 120-bp β2-microglobulin fragment was significantly expressed after 18 cycles of PCR and reached a plateau at 24 cycles. The 167-bp corresponding to MDR1 and the 291-bp corresponding to MRP products were undetectable up to 20 cycles. From 20 to 24 cycles, the three genes were amplified with comparable kinetics (yields of PCR products were 55.9% for MDR1, 56.7% for MRP and 51.7% for β2-microglobulin). Amplification was thus performed at 22 cycles.

This RT-PCR–ELISA assay markedly increased the sensitivity compared to classical detection, since MDR1 and MRP products were undetectable on ethidium bromide-stained agarose gels after 22 cycles of amplification. The intra-day reproducibility determined on the same cDNA sample ($n$ = 8) was 3.9% for MDR1 and 7.4% for MRP. The inter-day reproducibility (same cDNA sample) resulting from five independent experiments was 25.8% for MDR1 and 30.6% for MRP. In each series of analyses, an internal control is used which allows correction to be done. The internal control is an aliquot from a cDNA preparation obtained from a tumour specimen. The correction is done by comparing the result given by the internal control with the mean of repeated determinations on previous series.

### Description of tumoural parameters

The description of S phase fraction, p53, MDR1 and MRP is given in Table 2. Wide inter-patient variability was observed for all parameters: S phase varied within a 26-fold range, MDR1 expression within an 11-fold range, MRP within a 45-fold range and p53 protein from the limit of detection (0.002 ng $mg^{-1}$) up to 35.71 ng $mg^{-1}$ (two samples out of 90 had p53 concentrations below the limit of detection). ER and PR were positive in 71.8% and 65.9% of patients respectively.

Relationships between tumoural factors are reported in Table 3. P53 protein level was significantly different according to ER status (median twofold higher in ER-negative as compared to ER-positive, $P$ = 0.039). A weak but significant negative correlation was observed with MDR1: the higher the p53, the lower the MDR1

*© 2000 Cancer Research Campaign* 

**Table 2** Description of tumoural parameters

| | S phase (%) | p53 ($ng\ mg^{-1}$) | MDR1-mRNA (normalized/$\beta_2$-microglobulin) | MRP-mRNA (normalized/$\beta_2$-microglobulin) |
|---|---|---|---|---|
| *n* | 41 | 84 | 85 | 85 |
| Mean | 8.62 | 1.18 | 94 | 315 |
| Median | 6.38 | 0.13 | 83 | 242 |
| s.d. | 6.79 | 4.31 | 54 | 294 |
| 1st–3rd quartile | 3.02–14.12 | 0.06–0.28 | 60–114 | 75–429 |
| Min–max | 1.19–30.40 | ND–35.71 | 28–315 | 32–1452 |

ND, not detectable (< 0.002 ng $ml^{-1}$).

**Table 3** Tumoural parameters and relationships between them according to non-parametric tests

| | Histological grade | | | PR status | | ER status | | S Phase (%) | MRP | MDR 1 |
|---|---|---|---|---|---|---|---|---|---|---|
| | I | II | III | Pos. | Neg. | Pos. | Neg. | | | |
| p53 (ng $ml^{-1}$) | Median 0.09 | Median 0.20 | Median 0.13 | Median 0.13 | Median 0.12 | Median 0.11 | Median 0.21 | Spearman $r = 0.13$ | Spearman $r = -0.08$ | Spearman $r = -0.27$ |
| | | KW $P = 0.055$ | | MW $P = 0.91$ | | MW $P = 0.039$ | | $P = 0.43$ | $P = 0.45$ | $P = 0.015$ |
| MDR1 | Median 99 | Median 71 | Median 83 | Median 85 | Median 75 | Median 76 | Median 83 | Spearman $r = -0.21$ | Spearman $r = 0.19$ | |
| | | KW $P = 0.029$ | | MW $P = 0.67$ | | MW $P = 0.89$ | | $P = 0.20$ | $P = 0.078$ | |
| MRP | Median 290 | Median 150 | Median 258 | Median 218 | Median 272 | Median 226 | Median 259 | Spearman $r = -0.42$ | | |
| | | KW $P = 0.13$ | | MW $P = 0.66$ | | MW $P = 0.95$ | | $P = 0.006$ | | |
| S phase (%) | Median 2.54 | Median 6.38 | Median 15.94 | Median 4.66 | Median 11.41 | Median 4.02 | Median 13.07 | | | |
| | | KW $P = 0.010$ | | MW $P = 0.048$ | | MW $P = 0.002$ | | | | |
| ER status | 100% Pos. | 74.3% Pos. | 26.3% Pos. | Pos. 51 | 10 | | | | | |
| | | | | Neg. 5 | 19 | | | | | |
| | | $\chi^2$ $P < 0.001$ | | $\chi^2$ $P < 0.001$ | | | | | | |
| PR status | 95.5% Pos. | 68.6% Pos. | 26.3% Pos. | | | | | | | |
| | | $\chi^2$ $P < 0.001$ | | | | | | | | |

Pos., positive; Neg., negative; KW, Kruskal–Wallis test; MW, Mann–Whitney test; Spearman, Spearman rank correlation.

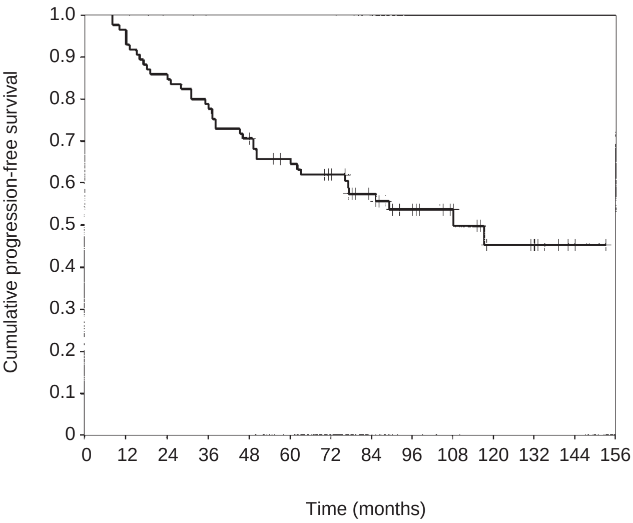

**Figure 2** Plot of cumulative progression-free survival according to Kaplan–Meier method. Survival was calculated from the date of surgery; end point was local recurrence and/or metastasis. A total of 85 patients were analysed (39 events observed). Vertical bars indicate the 46 censored observations expression ($P = 0.015$, $r = -0.27$). P53 was not linked to PR status, S phase fraction, MRP, and no clear relationship was observed according to the histological grade. MDR1-mRNA was significantly different according to the histological grade, with greater expression in grade I tumours ($P = 0.029$). MDR1 expression was not linked to the S phase fraction. Importantly, no relationship was observed between MDR1 and MRP expression. Neither MDR1 nor MRP was linked to ER or PR status. Unlike MDR1, MRP-mRNA was not different according to the tumour histological grade, and was significantly correlated with the S phase fraction: the greater the MRP-mRNA, the lower the S phase fraction ($P = 0.006$, $r = -0.42$).

### Survival analyses

At time of analysis, 39 patients had relapsed (12 local recurrences, 24 metastases, three patients with both metastases and local relapse). Progression-free survival is illustrated in Figure 2. The probability of 5-year progression-free survival was 0.64 with a median progression-free survival of 108 months. Analyses of prognostic factors are shown in Table 4. S phase fraction, MDR1 expression and MRP expression had no significant influence on progression-free survival. The above factors were also tested as categorial variables based on the median value (0 if lower than

 *© 2000 Cancer Research Campaign*

**Table 4** Univariate Cox analyses for progression-free and specific survival

| Co-variable | *n* | Progression-free survival | | Specific survival | |
|---|---|---|---|---|---|
| | | *P* | RR[a] | *P* | RR[a] |
| Histological grade | 76 | 0.048 | | 0.078 | |
| I (reference group) | 22 | | | | |
| II–III | 54 | | 2.43 | | 2.63 |
| Tumour size | 82 | 0.060 | | 0.15 | |
| T1 (reference group) | 14 | | | | |
| T2 | 54 | | 5.67 | | 7.35 |
| T3-T4 | 14 | | 4.44 | | 6.46 |
| Number of nodes involved | 85 | 0.81 | | 0.98 | |
| 1–3 (reference group) | 42 | | | | |
| 4–7 | 20 | | 0.84 | | 1.11 |
| > 7 | 23 | | 1.14 | | 1.04 |
| Age | 85 | 0.39 | 0.99 | 0.17 | 0.97 |
| ER status (0 : neg; 1 : pos) | 85 | 0.91 | 0.96 | 0.85 | 0.92 |
| PR status (0 : neg; 1 : pos) | 85 | 0.92 | 1.03 | 0.64 | 0.82 |
| S phase as logarithm 10 | 41 | 0.20 | 2.57 | 0.16 | 3.42 |
| S phase as categorial[b] | 41 | 0.15 | 2.24 | 0.13 | 2.88 |
| p53 as logarithm 10 | 84 | 0.076 | 1.40 | 0.38 | 1.24 |
| p53 as categorial[b] | 84 | 0.069 | 1.84 | 0.66 | 1.20 |
| MDR1 as logarithm 10 | 85 | 0.37 | 0.53 | 0.62 | 0.63 |
| MDR1 as categorial[b] | 85 | 0.29 | 0.71 | 0.25 | 0.62 |
| MRP as logarithm 10 | 85 | 0.82 | 1.09 | 0.63 | 0.79 |
| MRP as categorial[b] | 85 | 0.66 | 1.16 | 0.72 | 1.16 |

Neg, negative; Pos, positiive. [a] For any co-variable, the relative risk (RR) is equal to the risk of death of a patient presenting the value Xi divided by the risk of death of a patient presenting the value Xi-1. For categorial variables, RR represents the relative risk of death between the two classes of the variable. When RR>1, the risk of death rises when the variable increases; when RR<1, the risk of death decreases when the variable increases. [b]Variables analysed as categorial were recoded as 0 when < median and 1 when > median.

median, 1 if greater). However, when so doing, variables remain non-significant. A tendency suggested that the greater the p53 concentration, the shorter the progression-free survival ($P = 0.076$ as continuous and 0.069 as categorial variable, Table 4). The only significant predictor of progression-free survival was the histological grade ($P = 0.048$), with a relative risk of 2.43 (95% confidence interval 1.00–5.87) for grade II–III, as compared to grade I. The influence of clinical tumour size (T1 vs T2 vs T3–T4) was close to significance ($P = 0.060$).

Specific survival was analysed by considering the 25 breast cancer-related deaths (Figure 3). Probability of specific survival at 5 years was 0.81. Univariate Cox analyses revealed that S phase fraction, p53 protein level, MDR1 expression and MRP expression had no significant influence on specific survival (Table 4). As for progression-free survival, when tested as categorial variables (0 if lower than median, 1 if greater), these parameters remain non-significant. Also, the number of involved nodes was not a significant predictor of specific survival. The influence of histological grade was at the limit of significance ($P = 0.078$, Table 4).

### DISCUSSION

During the last decade, a plethora of clinical studies investigating the prognostic value of new tumoral markers in breast cancer has been published. Most of them focused on axillary node-negative patients in order to identify subgroups of at-risk patients who might benefit from adjuvant therapy (Gasparini et al, 1993). The scope of the present study was somewhat different. It aimed to

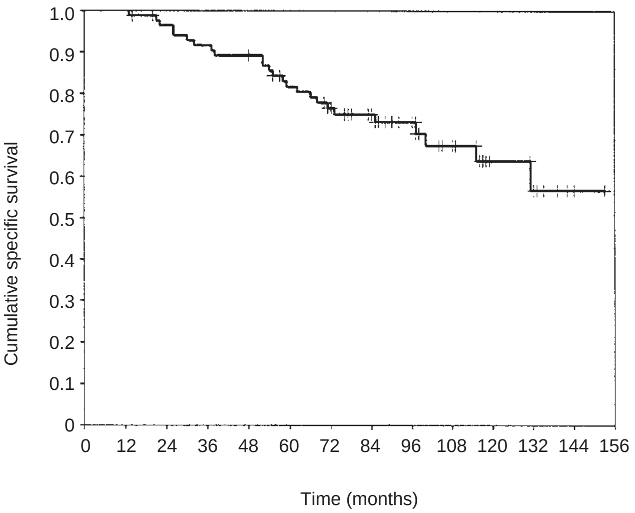

**Figure 3** Plot of cumulative specific survival according to Kaplan–Meier method. Survival was calculated from the date of surgery; end point was breast cancer-related death. A total of 85 patients were analysed (25 events observed). Vertical bars indicate the 60 censored observations determine whether tumoural factors considered to be relevant for drug efficacy could be helpful in predicting long-term outcome (7.8 years median follow-up) in node-positive breast cancer patients receiving anthracycline-based adjuvant chemotherapy. We thus developed and validated a RT-PCR–ELISA multiplex assay allowing simultaneous quantification of MDR1 and MRP mRNA since expression of MDR1 and MRP is known to be linked to anthracycline resistance phenotype (Filipits et al, 1996). Tumoural p53 was also investigated since p53 is involved in apoptosis control (Lowe et al, 1993; Shimamura and Fischer, 1996). In addition, it has been demonstrated that tumours expressing p53 wild-type gene contain a high proportion of apoptotic cells which regress after adriamycin treatment, whereas p53-mutated tumours contain few apoptotic cells and continue to grow (Lowe et al, 1994). To our knowledge, the present study is the first conducted in node-positive breast cancer patients receiving anthracycline-based adjuvant chemotherapy, with simultaneous measurement of p53, MDR1 and MRP. In addition, classical prognostic factors like histological grading, node involvement, S phase fraction, ER and PR were analysed.

As regards tumour size, histological grade and ER and PR status (Table 1), the present cohort of 85 patients is a representative subgroup of node-positive breast cancer patients (Muss et al, 1994). Also, the distribution of S phase fraction closely fits with data previously published (Muss et al, 1994).

In the present study, p53 mutations were indirectly estimated by measuring cellular retention of the p53 protein (immunoluminometric assay) which is markedly increased in p53-mutated cells (Raybaud-Diogene, 1996). Tumoural p53 exhibited tremendous inter-patient variability, with concentrations ranging from the limit of detection (< 0.002 ng $ml^{-1}$) up to 35.71 ng $ml^{-1}$ (median value at 0.13). P53 was significantly higher in ER-negative tumours as compared to ER-positive tumours (Table 3). P53 was not related to PR status, MRP, or to S phase fraction (Table 3). This latter result, obtained from a small group of 41 patients, contrasts with data from Allred (1993), Muss (1994), Iacopetta (1998) and Levesque (1998), who all reported a significant positive relationship between proliferation rate (S phase or Ki-67) and p53 expression

© 2000 Cancer Research Campaign or mutation. From the present set of 84 patients, p53 taken as continuous or dichotomous variable was not able to predict either long-term progression-free survival or specific survival, even though a tendency was observed suggesting that the greater the p53 concentration, the shorter the progression-free survival ($P$ = 0.076 as continuous and 0.069 as dichotomous variable, Table 4).

So far, the main study performed in node-positive breast cancer patients receiving adjuvant chemotherapy is that of Muss (1994) who performed immunohistochemistry on 394 tumours and demonstrated a significant prognostic value of p53 accumulation and of S phase fraction on overall survival (univariate analyses), but not on disease-free survival. Silvestrini et al (1996) investigated the role of p53 (immunohistochemistry) on 240 node-positive, ER-positive post-menopausal breast cancer patients receiving post-operative radiation plus tamoxifen: p53 expression was a significant indicator of relapse-free survival both in univariate and multivariate analysis including the number of nodes involved and labelling index. There is still a need for further evaluation of the value of p53 expression or mutations for predicting radio- or chemo-sensitivity in breast cancer patients.

Overexpression of MDR1 and MRP-related proteins has been demonstrated to be a major cause of the multidrug resistance phenomenon, which is characterized by an increased efflux of structurally unrelated drugs including anthracyclines, vinca-alkaloids, epipodophyllotoxins and taxanes (Lautier et al, 1996). In contrast to the abundance of published MDR1 studies, MRP expression has so far been poorly investigated in breast cancer (Nooter et al, 1997; Beck et al, 1998). In the present study, an original RT-PCR–ELISA multiplex assay allowing simultaneous analysis of MDR1 and MRP was developed, validated and applied on 85 tumour specimens. Inter-patient variability for MRP was greater than that observed for MDR1 (45-fold and 11-fold range respectively, Table 2). No significant relationship was demonstrated between MDR1 and MRP expression ($P$ = 0.078, Table 3). This finding corroborates the work by Filipits (1996) on 134 tumours and that of Dexter (1998) on 74 tumour specimens, but contrasts with the data of Beck (1998) who reported a significant correlation on 62 primary breast cancers. Of clinical relevance are the recent data of Dexter (1998) who used competitive RT-PCR to measure MDR1 and MRP expression and demonstrated that expression of MDR1 was extremely low as compared to MRP. In line with our data, Dexter et al (1998) showed that MDR1 and MRP expression were independent of ER and PR status. In our study, MDR1 expression was significantly greater in histological grade I tumours as compared to grade II–III (Table 3). Noteworthy, a significant negative correlation was demonstrated between MRP expression and S phase fraction (Table 3). This observation may explain the fact that breast tumours with a high proliferation rate exhibit higher response rates to preoperative chemotherapy than tumours with a low proliferation rate (Remvikos et al, 1989). In addition, we observed a weak but significant negative correlation between MDR1 expression and p53 concentrations (Table 3). However, based on the previously reported stimulation of MDR1 promotor gene by a mutant p53 protein (Chin, 1992), an inverse result would have been expected.

Analysed both as a continuous or a dichotomous variable, neither MDR1 nor MRP expression was related to progression-free survival which is dependent on the efficiency of anthracycline-based adjuvant therapy (Table 4). So far, the value of MDR1 and/or MRP expression in predicting treatment efficacy in breast cancer patients has not been clearly established (Linn, 1995; Nooter, 1997). The consensus recommendations recently published for measuring MDR1/P-glycoprotein expression in clinical studies (Beck et al, 1996) will probably help to clarify the role of MDR1 expression in predicting treatment outcome in breast cancer patients. Using the previously published classification of Scarff (1968) and Bloom (1957), histological grading was presently scored taking into account the degree of differentiation, nuclear polymorphism and the mitotic index. In the present long-term prognostic study, the only significant factor was the histological grading, linked to progression-free survival ($P$ = 0.048, Table 4); a tendency was observed towards specific survival ($P$ = 0.078, Table 4).

In conclusion, the present study provides a new tool for simultaneous measurement of MDR1 and MRP expression in tumour specimens. Tannock (1998) recently pointed out the need to individualize treatment in order to improve the effectiveness of chemotherapy and thus survival for breast cancer patients receiving adjuvant chemotherapy. We hope the present MDR1-MRP assay may contribute to better evaluation of such a strategy.

### REFERENCES

Abbaszadegan MR, Futscher BW, Klimecki WT, List A and Dalton WS (1994) Analysis of multidrug resistance-associated protein (MRP) messenger RNA in normal and malignant hematopoietic cells. *Cancer Res* **54**: 4676–4679

Allred DC, Clark GM, Elledge R, Fuqua SAW, Brown RW, Chamness GC, Osborne CK and McGuire WL (1993) Association of p53 protein expression with tumor cell proliferation rate and clinical outcome in node-negative breast cancer. *J Natl Cancer Inst* **85**: 200–206

Barrand MA, Heppell-Parton AC, Wright KA, Rabbits PH and Twentyman PR (1994) A 190-kilodalton protein overexpressed in non-p-glycoprotein-containing multidrug resistant cells and its relationship to the MRP gene. *J Natl Cancer Inst* **86**: 110–117

Beck WT, Grogan TM, Willman C and 28 others (1996) Methods to detect P-glycoprotein-associated multidrug resistance in patients' tumors: consensus recommendations. *Cancer Res* **56**: 3010–3020

Beck J, Bohnet B, Brügger D, Bader P, Dietl J, Scheper RJ, Kandolf R, Liu C, Niethammer D and Gekeler V (1998) Multiple gene expression analysis reveals distinct differences between $G_2$ and $G_3$ stage breast cancers and correlations of $PKC_n$ with $MDR_1$, MRP and LRP gene expression. *Br J Cancer* **77**: 87–91

Bloom HJ and Richardson WW (1957) Histological grading and prognosis in breast cancer. *Br J Cancer* **11**: 359–377

Castillo L, Milano G, Santini J, Demard F and Pierrefite V (1997) Analysis of retinoic acid receptor β expression in normal and malignant laryngeal mucosa by a sensitive and routine applicable reverse transcription-polymerase chain reaction enzyme-linked immunosorbent assay method. *Clin Cancer Res* **3**: 2137–2142

Chin KV, Ueda K, Pastan I and Gottesman MM (1992) Modulation of activity of the promotor of the human MDR1 gene by ras and p53. *Science* **255**: 459–462

Chomczynski P and Sacchi N (1987) Single-step method of RNA isolation by acid guanidinium thiocyanate-phenol-chloroform extraction. *Annal Biochem* **162**: 156–159

Dexter DW, Reddy RK, Geles KG, Bansal S, Myint MA, Rogakto A, Leighton JC and Goldstein LJ (1998) Quantitative reverse transcriptase-polymerase chain reaction measured expression of MDR1 and MRP in primary breast carcinoma. *Clin Cancer Res* **4**: 1533–1542

Early Breast Cancer Trialists' Collaborative Group (1992) Systemic treatment of early breast cancer by hormonal, cytotoxic, or immune therapy: 133 randomised trials involving 31 000 recurrences and 24 000 deaths among 75 000 women. *Lancet* **339**: 71–85

Early Breast Cancer Trialists' Collaborative Group (1998) Polychemotherapy for early breast cancer: an overview of the randomized trials. *Lancet* **352** 930–942

Filipits M, Suchomel RW, Dekan G, Haider K, Valdimarsson G, Depisch D and Pirker R (1996) MRP and $MDR_1$ gene expression in primary breast carcinoma. *Clin Cancer Res* **2**: 1231–1237

Gasparini G, Pozza F and Harris AL (1993) Evaluating the potential usefulness of new prognostic and predictive indicators in node-negative breast cancer patients. *J Natl Cancer Inst* **85**: 1206–1219

© 2000 Cancer Research Campaign

Iacopetta B, Grieu F, Powell B, Soong R, McCaul K and Seshadri R (1998) Analysis of p53 gene mutation by polymerase chain reaction-single-strand conformation polymorphism provides independent prognostic information in node-negative breast cancer. *Clin Cancer Res* **4**: 1597–1602

Lautier D, Canitrot Y, Deeley RG and Cole SPC (1996) Multidrug resistance mediated by the multidrug resistance protein (MRP) gene. *Biochem Pharmacol* **52**: 967–977

Levesque MA, Yu H, Clark GM and Diamandis EP (1998) Enzyme-linked immunoabsorbent assay-detected p53 protein accumulation: a prognostic factor in a large breast cancer cohort. *J Clin Oncol* **16**: 2641–2650

Linn SC, Giaccone G, Van Diest PJ, Blokhuis WMD, Van der Valk P, Van Kalken CK, Kuiper CM, Pinedo HM and Baak JPA (1995) Prognostic relevance of P-glycoprotein expression in breast cancer. *Annal Oncol* **6**: 679–685

Lowe SW, Ruley HE, Jacks T and Housman DE (1993) P53-dependent apoptosis modulates the cytotoxicity of anticancer agents. *Cell* **74**: 957–967

Lowe SW, Bodis S, McClatchey A, Remington L, Ruley HE, Fisher DE, Housman DE and Jacks T (1994) P53 status and the efficacy of cancer therapy in vivo. *Science* **266**: 807–810

Muss HB, Thor AD, Berry DA, Kute T, Liu ET, Koerner F, Cirrincione CT, Budman DR, Wood WC, Barcos M and Henderson IC (1994) C-erb B-2 expression and response to adjuvant therapy in women with node-positive early breast cancer. *N Engl J Med* **330**: 1260–1266

Namer M, Ramaioli A, Fontana X, Etienne MC, Hery M, Jourlait A, Milano G, Frenay M, François E and Lapalus F (1991) Prognostic value of total cathepsin D in breast tumors. A possible role in selection of chemoresistant patients. *Breast Cancer Res Treat* **19**: 85–93

Noonan KE, Beck C and Holzmayer TA (1990) Quantitative analysis of $MDR_1$ (multidrug resistance) gene expression in human tumors by polymerase chain reaction. *Proc Natl Acad Sci USA* **87**: 7160–7164

Nooter K, Brutel de la Riviere G, Look MP, Van Wingerden KE, Henzen-Logmans SC, Scheper RJ, Flens MJ, Klijn JGM, Stoter G and Foekens JA (1997) The prognostic significance of expression of the multidrug resistance associated protein (MRP) in primary breast cancer. *Br J Cancer* **76**: 486–493

Pastan I and Gottesman MM (1987) Multiple-drug resistance in human cancer. *N Engl J Med* **316**: 1388–1393

Raybaud-Diogène H, Tétu B, Morency R, Fortin A and Monteil RA (1996) P53 overexpression in head and neck squamous cell carcinoma: review of the literature. *Oral Oncol, Eur J Cancer* **32B**: 143–149

Remvikos Y, Beuzedoc P, Zajdela A, Voillemot N, Magdelenat H and Pouillart P (1989) Correlation of pretreatment proliferative activity of breast cancer with the response to cytotoxic chemotherapy. *J Natl Cancer Inst* **81**: 1383–1387

Romain S, Formento JL, Guirou O, Francoual M, Milano G and Martin PM (1994) Determination of oestrogen receptors by enzyme immunoassay. Technical differences between laboratories and their consequences. *Eur J Cancer* **30**: 740–746

Scarff RW and Torloni H (1968) Histological typing of breast tumors. Geneva: *World Health Organization*: 13–20

Shimamura A and Fisher DE (1996) P53 in life and death. *Clin Cancer Res* **2**: 435–440

Silvestrini R, Benini E, Veneroni S, Daidone MG, Tomasic G, Squicciarini P and Salvadori B (1996) P53 and bcl-2 expression correlates with clinical outcome in a series of node-positive breast cancer patients. *J Clin Oncol* **14**: 1604–1610

Tannock IF (1998) Conventional cancer therapy: promise broken or promise delayed. *Lancet* **351** (suppl II): 9–16

*© 2000 Cancer Research Campaign*
